# Population Genomics Reveals Demographic History and Genomic Differentiation of *Populus davidiana* and *Populus tremula*

**DOI:** 10.3389/fpls.2020.01103

**Published:** 2020-07-22

**Authors:** Zhe Hou, Ang Li

**Affiliations:** ^1^Key Laboratory of Southwest China Wildlife Resources Conservation (Ministry of Education), College of Life Science, China West Normal University, Nanchong, China; ^2^State Key Laboratory of Tree Genetics and Breeding, Chinese Academy of Forestry, Beijing, China

**Keywords:** *Populus davidiana*, *Populus tremula*, population genomics, genetic diversity, demographic history, genetic adaptation

## Abstract

Forest trees can increase our understanding of how evolutionary processes drive the genomic landscape and understand speciation due to the majority of forest trees being distributed widely and able to adapt to different climates and environments. *Populus davidiana* and *Populus tremula* are among the most geographically widespread and ecologically important tree species in Northern Hemisphere. Whole-genome resequencing data of 41 individuals of *P. davidiana* and *P. tremula* throughout Eurasia was conducted, finding that genetic differentiation was evident between the two species, the *F_ST_* values between *P. davidiana* and *P. tremula* was 0.3625. The ancestors of the two aspen diverged into *P. davidiana* and *P. tremula* species approximately 3.60 million years ago (Mya), which was in accordance with the rapid uplift of Qinghai–Tibet Plateau (QTP) around the Miocene/Pliocene boundary. The two species experienced a considerable long-term bottleneck after divergence, with population expansion beginning approximately 20,000 years ago after the end of the last glacial maximum. Although the majority of regions of genomic differentiation between the two species can be explained by neutral evolutionary processes, some outlier regions have also been tested that are significantly influenced by natural selection. We found that the highly differentiated regions of the two species exhibited significant positive selection characteristics, and also identified long-term balancing selection in the poorly differentiated regions in both species. Our results provide strong support for a role of linked selection in generating the heterogeneous genomic landscape of differentiation between *P. davidiana* and *P. tremula*. These results provide the detailed and comprehensive genomic insights into genetic diversity, demography, genetic burden, and adaptation in *P. davidiana* and *P. tremula*.

## Introduction

Increasing our understanding of how evolutionary processes drive the genomic landscape of variation is fundamental to a better understanding of the genomic consequences of speciation. Understanding how and why genomes diverge during speciation has received considerable attention in evolutionary biology research. (Noor and Bennett, 2009; Nachman and Payseur, 2012; Nosil and Feder, 2012; Strasburg et al., 2012; Seehausen et al., 2014). Generally, a combination of evolutionary factors has an effect on the divergence during the process of speciation, such as demographic fluctuations, genetic drift, mutation, recombination rates, genetic hitchhiking, background selection and migration all play important roles to shape the heterogeneity of species divergence (Wang et al., 2016). In accordance with strict neutral theory, the mechanisms of genetic differentiation are the result of changing allele frequencies due to genetic drift and novel mutations (Hellmann et al., 2005). Demographic factors can trigger differentiation throughout the genome deviating from strict neutrality through a change in the effective population size such as population expansion or bottlenecks (Li and Durbin, 2011). Dramatic climatic oscillations and historical geology especially can shape the geographic location patterns of numerous plant groups and triggered population differentiation and even speciation (Sanna et al., 2008). Demographic fluctuations and genetic drift cause variation throughout the genome (Luikart et al., 2003). Nevertheless, Darwinian or natural selection affects only genes that provide important functional information. For example, both positive and purifying selection can cause genetic variation in reproductive isolation or ecological specialization loci that influence the fitness and respective phenotypes of an organism (Via, 2009). Recombination and mutation rates that affect important functional architecture of the entire genome are also essential evolutionary factors that determine the heterogeneity of genomic divergence (Noor and Bennett, 2009; Nachman and Payseur, 2012). In general, a combination of evolutionary factors affects the patterns of overall genomic variation during the process of population differentiation, such as demographic fluctuations, genetic drift, mutation, recombination rates, genetic hitchhiking, background selection and migration, all performing important roles to shape the heterogeneity of genomic divergence (Wang et al., 2016). However, disentangling the relative importance of these evolutionary forces when interpreting patterns of genomic divergence remains a challenge in speciation genetics.

A growing quantity of genome-wide data are becoming available with the development of high-throughput sequencing technology, and intense research activity has resulted in the discovery of substantial patterns of genetic variation and population divergence among multiple related species with considerably increased accuracy (Turner et al., 2005; Ellegren et al., 2012; Feulner et al., 2015). A universal interpretation of genetic differentiation from the overall genome suggests different levels of gene flow. A number of sites associated with reproductive isolation usually have higher levels of genetic differentiation, also commonly referred to as “genomic islands”, whereas lower levels of variation are often observed in other sites across the genome due to gene flow (Nosil et al., 2009). However, other studies have indicated that highly differentiated regions in the genome are incidental rather than directly related to ecological speciation. The authors have argued that highly differentiated regions occur because linked selection (positive and purifying selection) substantially reduces genetic diversity by removing neutral polymorphism and increases genome divergency, especially in regions with low rates of recombination (Cruickshank and Hahn, 2014). Furthermore, long-term balancing selection increases variability within a population resulting in low genetic differentiation between species (Charlesworth et al., 1995). It is now apparent that the different forms of natural selection (positive, purifying and balancing selection) alone are enough to shape the different patterns of genomic differentiation (Turner et al., 2005). Finally, genomic divergence deviating from the strict neutrality model can also be shaped by neutral forces, such as demographic fluctuations, mutation and stochastic genetic drift (Nosil et al., 2009; Campagna et al., 2015). In general, the hypotheses listed above are not mutually exclusive and exhaustive examination of these hypotheses requires detailed information on the speciation process, such as the timing of speciation, the geographic and demographic context in which it occurred (Nosil and Feder, 2012).

Forest trees are an excellent resource for understanding speciation and genome variation patterns due to the majority of them are distributed widely and can adapt to variations in climate and the environment without any anthropogenic influence, and harbor a wealth of genetic variation (Neale and Antoine, 2011). *Populus davidiana* Dode and *Populus tremula* L. are two of the most ecologically important and geographically widespread tree species of the Northern Hemisphere. Both are keystone species, display rapid growth, with high tolerance to environmental stresses and long-distance pollen and seed dispersal *via* wind (Müller et al., 2009). In addition, they both harbor among the highest level of intraspecific genetic diversity reported in plant species so far (Ingvarsson, 2008). Based on their morphological similarity and close phylogenetic relationship, they are considered to be sister species, or less commonly, conspecific subspecies (Eckenwalder, 1977; Wang et al., 2014). They can readily cross and artificial hybrids usually show high heterosis (Hamzeh et al., 2009). *P. davidiana* and *P. tremula* are deciduous, obligated outcrossing trees in section *Leuce* (*Populus*, *Salicaceae*) and are keystone taxa in boreal forest communities (Joshi et al., 2011). These congeners share similar ecological and latitudinal distribution ranges but reside on different continents (Morin et al., 2015). *P. davidiana* is mainly distributed in mainland China, on the Korean peninsula and in Japan. *P. tremula* occurs throughout Europe, Siberia and Xinjiang, China. The taxonomy of these two aspens has been controversial with respect to their extreme morphological congruence with only minor differences in leaf shape (Löve and Löve, 1975). Furthermore, previous phylogenetic analyses of *Populus* elucidated close genetic affinity among these two aspens (Hamzeh et al., 2009). For example, based on the phylogeny of *Populus* reconstructed using 24 single copy nuclear loci, *P. davidiana* and *P. tremula* clustered in a single clade within section *Leuce* with a relatively high bootstrap value or posterior probability (Wang et al., 2014). A recent study based on a handful of chloroplast loci and morphological analysis also suggests that *P. davidiana* and *P. tremula* are sister species (Zong et al., 2019). Earlier phylogenetic studies have revealed that the uplift of the Qinghai–Tibetan Plateau and the associated climate oscillations may have driven the divergence between *P. tremula* and *P. davidiana* (Du et al., 2015).

Both *P. davidiana* and *P. tremula* have wide geographic distribution, high intraspecific polymorphism, adaptability to different environments, phenotypic diversity, combined with a relatively small genome size. Consequently, *P. davidiana* and *P. tremula* represent excellent models for understanding how different evolutionary forces have sculpted the variation patterns in the genome during the process of speciation. In the present study, next generation sequencing (NGS) was used to analyze 41 P*. davidiana* and *P. tremula* trees to explore population structure, estimate population divergence time points, identify the historical demographic processes and infer the overall patterns of genomic divergence. This study provides insights into the evolutionary history and genetic diversity of the two species, in addition to describing examples of the mechanisms by which the species can adapt to regions with variations in climate and also provides the important reference value for understanding the mechanism of the formation of the geographical distribution patterns of other plant populations in Eurasian.

## Materials and Methods

### Sample Collection, Whole-Genome Resequencing and Genotype Calling

A total of 20 individual of *P. davidiana* and 21 individual of *P. tremula* (**Table S1**) were collected and sequenced. The genomic DNA from all specimens was extracted from the leaves in accordance with a CTAB method (Pahlich and Gerlitz, 1980). A paired-end sequencing libraries with an insert size of 600 bp were constructed according to the Illumina library preparation protocol for every *P. davidiana* and *P. tremula* specimen and sequencing performed from high quality DNA based on the standard Illumina HiSeq 2000 platform protocol with an expected target coverage of 30×. The raw sequence data reported in this paper have been submitted to the Genome Sequence Archive (Wang et al., 2017) at the BIG Data Center, Beijing Institute of Genomics (BIG), Chinese Academy of Sciences, under accession numbers CRA001674 and CRA001683. CRA001674 and CRA001683 are publicly accessible at http://bigd.big.ac.cn/gsa. Prior to read mapping, we used Trimmomatic (Lohse et al., 2012) to remove adapter sequences and to trim low quality bases from the start or the end of reads (base quality ≤20). If the processed reads were shorter than 36 bases after trimming, the entire reads were discarded. After quality control, the BWA-MEM algorithm (Li et al., 2009) was used with parameters: “-t 8 -k 32 -M -R” to map all clean data to the *Populus trichocarpa* Torr. and A. Gray ex. Hook. reference genome, version 3 (Tuskan, 2006). SAMtools (Li et al., 2009) was used to sort the resulting reads after mapping, we then used RealignerTargetCreator and IndelRealigner (Depristo et al., 2011) to correct for the mis-alignment of bases in regions around insertions and/or deletions (indels). Duplicated reads were removed using MarkDuplicates available in the Picard application (http://broadinstitute.github.io/picard). Additionally, we further discarded site types that likely cause mapping bias based on three criteria: Where total read coverage was particularly low (<100) or extremely high (>1,200) across all *P. davidiana* and *P. tremula* samples reads or sites were completely filtered out; reads or sites that included >20 mapping quality scores of zero within the whole sample were discarded. These quality control steps resulted in only high quality reads being kept.

After filtering, we implemented two complementary strategies for downstream analysis (**Figure S1**). ANGSD v0.928 (Korneliussen et al., 2014) is a classic software package for the analysis of genome sequencing data, which was employed to estimate the site frequency spectrum (SFS), but not to call genotypes. Low-quality data were filtered out, reads that had a mapping quality <30 and bases with a quality score <20 were not considered. The SAMTools genotype likelihood model (Li et al., 2009) with the parameter -doSaf implemented to estimate SFS probability for calculating all population genetic statistics was employed. HaplotypeCaller and GenotypeGVCFs modules in GATK v3.7.1 (Depristo et al., 2011) were used to perform accurate genotype and SNP calls. In order to minimize genotype calling bias and to retain high-quality single nucleotide polymorphisms (SNPs), we further performed several filtering steps:(1) SNPs that overlapped with sites not passing all previous filtering criteria were removed; (2) only bi-allelic SNPs with a distance of at least 5 bp away from any indels were retained; (3) genotypes with read depth (DP) <5 or with genotype quality score (GQ) <10 were treated as missing, and we then removed all SNPs with a genotype missing rate >10%.

### Phylogenetics, Population Structure and Principal Components Analysis

we used the program NGSadmix, which is based on genotype likelihoods to directly estimate individual admixture proportions from next generation sequencing data (Skotte et al., 2013) to infer population genetic structure in *P. davidiana* and *P. tremula*, and sites with less than 10% of their data missing were used, the number of coancestry clusters (K) ranging from 1 to 6. Principal component analysis (PCA) was performed on all SNPs using the smartpca program in PCAngsd software (http://www.popgen.dk/software/index.php/PCAngsd). A Tracy–Widom test was used to determine the significance level of eigenvectors. The phylogenetic tree was constructed using neighbor-joining (NJ) with TreeBest software (http://treesoft.sourceforge.net/treebest.shtml), with *P. tremuloides* Michx. used as an outgroup. We downloaded the data from the Short Read Archive (SRA) at NCBI and the accession numbers is SRP065065.

### Demographic History of *P. davidiana* and *P. tremula*

We used coalescent simulations applying the composite likelihood method implemented in Fastsimcoal 2.6.1 software (Excoffier et al., 2013) to infer demographic parameters of the *P. davidiana* and *P. tremula* species based on the site frequency spectrum. Allele frequencies in the 41 samples were calculated using the realSFS module in ngsTools software so as to construct the required two-dimensional joint site frequency spectrum (2D-SFS), which was estimated with 100,000 coalescent simulations in each model. Alternative models of historical events were fitted to the joint site frequency spectra data. All parameter estimates were global ML estimates from 50 independent fastsimcoal2.1 runs, with 100,000 simulations per likelihood estimation (-n100,000, -N100,000) and 40 cycles of the likelihood maximization algorithm. The best model was identified through the maximum value of likelihoods and Akaike’s information criterion (AIC); simulated datasets were compared with the observed site frequency spectra to evaluate the fit of the best demographic model (Excoffier et al., 2013). In the calculation, we used the mutation rate of 2.5×10^−9^ per site per year and a generation time of 15 years to convert the coalescent scaled time to absolute time in years (Koch et al., 2000).

We used Multiple Sequentially Markovian Coalescent approach (MSMC v2) (Schiffels and Durbin, 2014) to infer historical patterns of effective population sizes changes of *P. davidiana* and *P. tremula* species. Prior to performing the calculation, all segregating sites within each population were phased and imputed using Beagle software (Browning et al., 2018). We assumed a mutation rate of 2.5×10^−9^ per site per year and a generation time of 15 years when converting the scaled time and effective population size to actual time and size (Tuskan, 2006).

### Genetic Differentiation and Selective Signals in *P. davidiana* and *P. tremula*

The polymorphism levels in each group were quantified by pairwise nucleotide diversity (θπ) and the genetic differentiation in two populations was quantified by pairwise *F_ST_*. Both hp and *F_ST_* were calculated by a sliding window method (100 kb windows sliding in 10 kb steps). Variants were filtered when the minor allele frequency was less than 5% and the missing genotypes frequency was more than 50%. For comparing groups (groups 1 and 2), the regions with maximum *F_ST_* values (top 5%) and minimum θπ_1_/θπ_2_ (top 5%) were identified as selected regions for group 1, and vice versa.

### Population Genetic Analysis and Molecular Signatures of Selection in Outlier Regions

To assess the occurrence of selection in outlier windows displaying either exceptionally high or low differentiation, multiple population genetic parameters of the two unions of outer regions were compared with the remaining portion of the genome by a variety of additional population genetic statistics in both species. Firstly, we used ANGSD to estimate sample allele frequency probabilities between populations of the *P. davidiana* and *P. tremula* over non-overlapping 10 Kbp windows for calculating Fay & Wu’s H (Fay and Wu, 2000), Fu and Li’s D (Fu and Li, 1993) and θπ. Secondly, to evaluate levels of LD within each 10 Kbp window, the correlation coefficients (r^2^) between SNPs with pairwise distances larger than 1 Kbp were calculated using VCFtools v0.1.12b (Danecek et al., 2011). And we used FastEPRR software (Gao et al., 2016) to calculated recombination rates (ρ) over a window size of 10,000 bp. Finally, we used the program ngsStat (Fumagalli et al., 2014) to calculate several additional measures of genetic differentiation: (1) the proportion of inter-specific shared polymorphisms among all segregating sites; (2) with *P. tremuloides* as an outgroup, the proportion of fixed differences that is caused by derived alleles fixed in either *P. davidiana* and *P. tremula* was calculated among all segregating sites; (3) the relative node depth (RND), calculated by dividing the dxy of the *P. davidiana* and *P. tremula* species by the dxy between the *P. davidiana* population and *P. tremuloides*. For all population genetic parameters, Wilcoxon ranked-sum tests were used to examine the significance of differences between outlier regions and the remainder of the genome. (4) dxy, which was calculated based on the posterior probability of the sample allele frequency at each locus and was then averaged over each 10 Kbp window.

## Results

A total of 20 P*. davidiana* and 21 P*. tremula* whole-genome resequenced data were generated for downstream analysis. The genomes of the two aspen and *P. trichocarpa* are highly conserved (Pakull et al., 2009), such that more than 88.43% (**Table S1**) of all *P. davidiana* and *P. tremula* sequences can be mapped to the reference genome of *P. trichocarpa* (Tuskan, 2006) following a quality control process. The mean coverage of each site reached 28.6 in mapped reads of *P. davidiana* and *P. tremula* samples (**Table S1**). After filtration and strict quality control, a total of 5,183,105 and 6,162,812 SNPs high-quality SNP sites were obtained for across the 21 P*. tremula* samples and 20 P*. davidiana* samples, respectively.

### Population Structure

**Figure 1:**
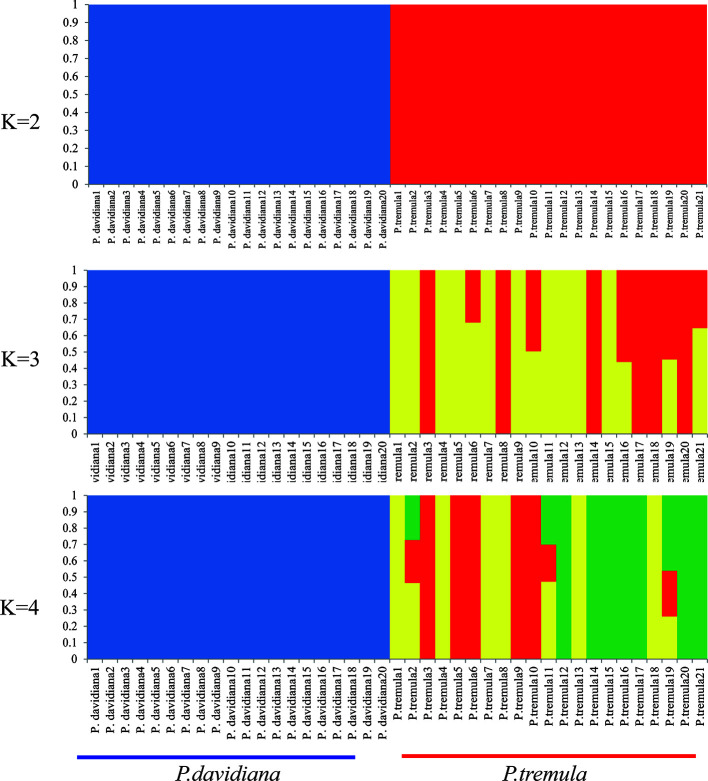
Genetic structure of *P. davidiana* and *P. tremula* inferred using NGSadmix.

**Figure 2:**
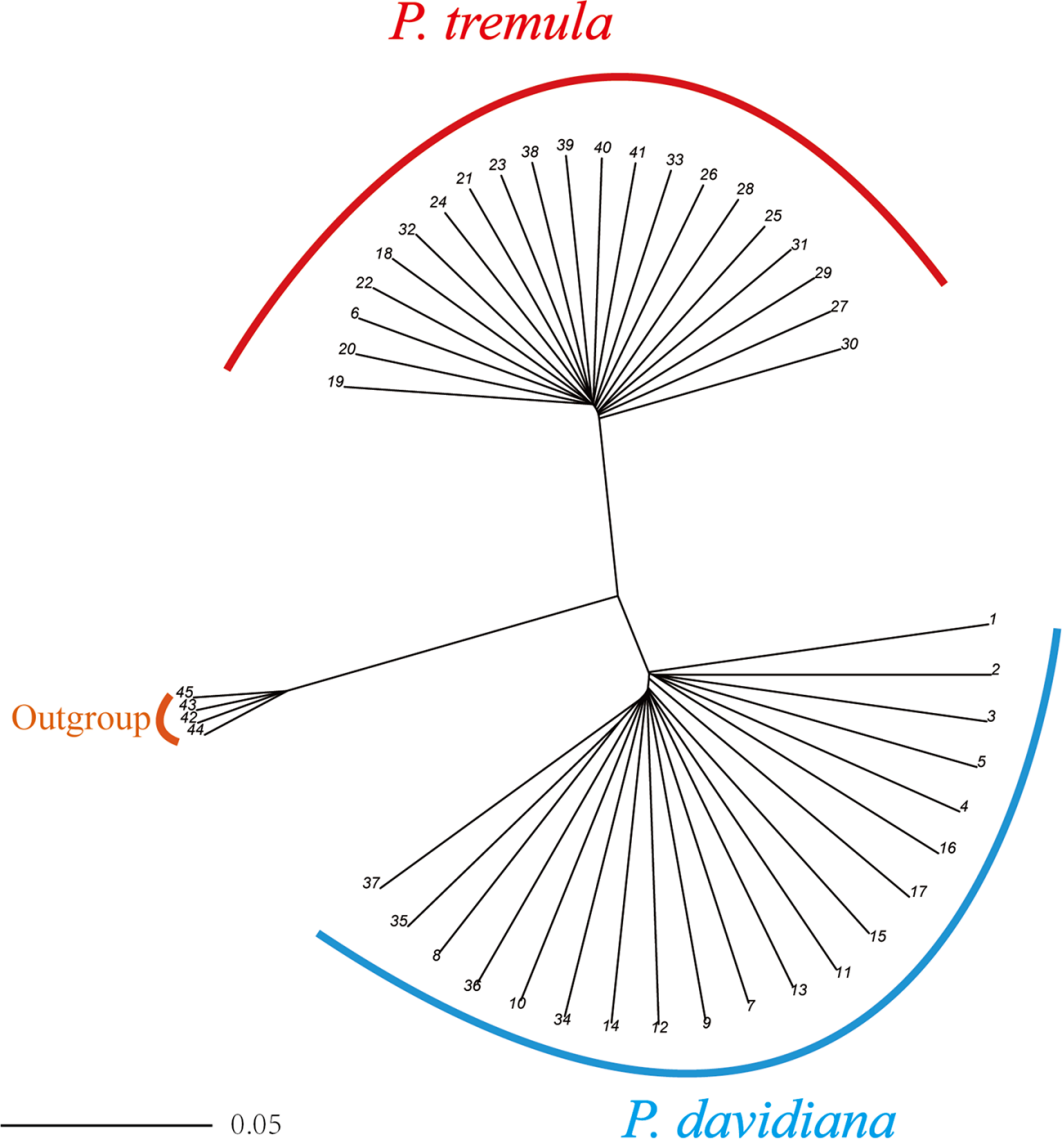
A rooted neighbor-joining tree constructed from the allele-shared matrix of SNPs among the *P. davidiana* and *P. tremula*, with the *P. tremuloides* as an outgroup.

**Figure 3:**
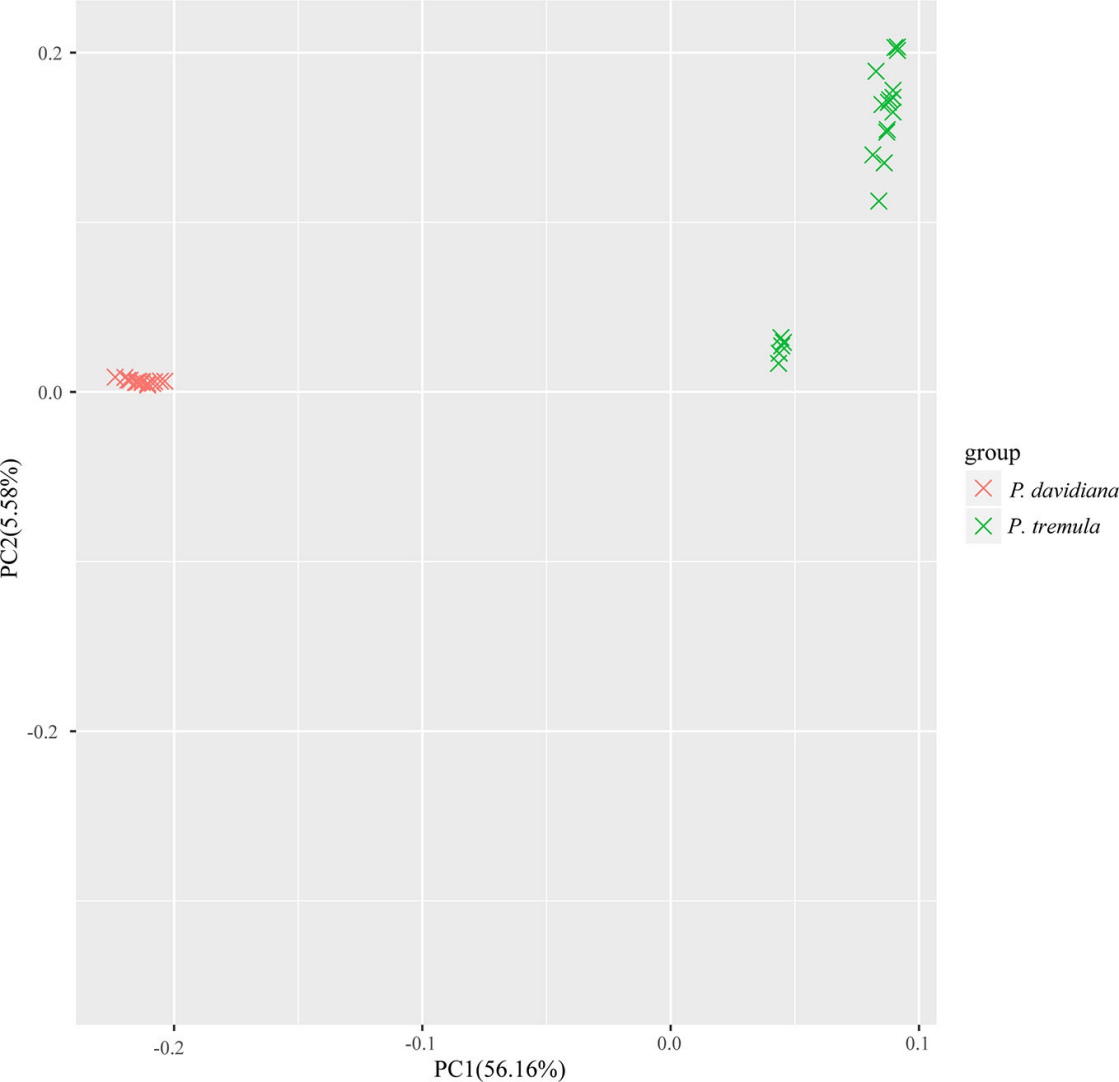
Principal component analysis (PCA) plot based on genetic covariance.

We used NGSadmix to infer individual ancestry based on genotype likelihoods, which takes the uncertainty of genotype calling into account. It clearly sub-divided all sampled individuals into two species-specific groups when the number of clusters (K) was 2. Further population sub-structuring was observed in *P. tremula* population when K = 3, most individuals of *P. tremula* were inferred to be a mixture of two genetic components, showing slight clinal variation with latitude. No further structure was found when K = 4 (**Figure 1**). A neighbor-joining tree was also constructed using *P. tremuloides* as an outgroup that further supported these patterns, with different geographical locations from the *P. davidiana* and *P. tremula* reflecting the grouping of populations (**Figure 2**). A principal component analysis (PCA) further supported these results. We found that the first two components explained 56.16 and 5.58% of total genetic variance according to a Tracy–Widom test, respectively (**Figure 3**). Among the total number of polymorphisms in the two species, fixed differences between *P. davidiana* and *P. tremula* accounted for 2.6%, whereas 12.3% of polymorphisms were shared between species, with the remaining polymorphic sites being private in either of the two species (**Figure S2**).

### Demographic Histories

**Figure 4:**
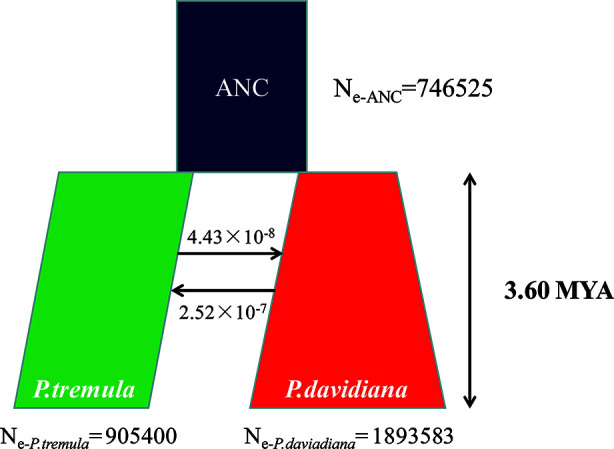
Best-fitting model inferred demographic histories and differentiation mode for *P. davidiana* and *P. tremula* implemented by fastsimcoal 2.6.1.

A coalescent simulation-based method was employed to infer demographic histories of *P. davidiana* and *P. tremula*. Eighteen different models were formulated to simulate the past population histories of *P. davidiana* and *P. tremula* that differed in terms of: (A) AsymmetricMigration without population expansion; (B) NoMigration without population expansion; (C) AsymmetricMigration with population expansion; (D) complex model, including a bottleneck in N; (E) complex model, including a bottleneck in S (**Figure S3**, **Table 1**). The most appropriate model was one of complex isolation-with-migration, after the two species diverged, *P. davidiana* experienced exponential growth, whereas a stepwise population size change occurred in *P. tremula* (**Figure 4**). A detailed effective population size, differentiation time point and gene flow of *P. davidiana* and *P. tremula* is displayed in **Table 2**, which also presents the 95% confidence interval (CIs) for the related parameters. The ancestors of the two aspen diverged into *P. davidiana* and *P. tremula* populations approximately 3.60 million years ago (Mya) (bootstrap range [BR]: 3.58–3.65 Mya). The current effective population sizes (Ne) of *P. tremula* (N_e-_*_P. tremula_*) and *P. davidiana* (N_e-_*P. _davidiana_*) are 905,400 (BR: 891,235-912,578) and 1,893,583 (BR:1,883,565–1,902,325), respectively. The effective population sizes of the two species are all significantly higher than their common ancestor (N_e-ANC_ = 746,525 [721,632–756,985]). The migration rate (m) is also clear among the two species, the lowest generation migration rate (m) between *P. davidiana* and *P. tremula* (4.43 × 10^−8^ and 2.52 × 10^−7^), not an accident due to the large geographical distance between the two populations.

**Table 1:** Relative likelihood of the different models.

Model	Max(log10(Lhoodi)^a^	AIC_i_^b^	Δi^b^	Model normalizedrelative likelihood (w_i_)^b^
Model 1	−766,690,638.2	353,302,140.1	4,348,409.527	~0
Model 2	−767,779,149.8	356,589,753.3	3,698,253.525	~0
Model 3	−769,356,470.5	354,589,522.2	2,635,452.236	~0
Model 4	−703,841,628.9	354,965,820.5	0	~1
Model 5	−739,200,638.81	355,289,547.6	2,015,896.025	~0
Model 6	−742,253,555.2	356,985,172.1	215,486.026	~0
Model 7	−742,377,897.5	359,863,214.1	1,548,753.025	~0
Model 8	−741,802,181.6	354,879,632.3	698,541.024	~0
Model 9	−720,729,162.1	352,308,124.1	1,523,698.014	~0
Model 10	−737,524,429.4	353,021,548.3	1,478,523.021	~0
Model 11	−737,955,129.6	358,796,852.5	4,589,632.027	~0
Model 12	−740,117,914.8	353,258,963.5	2,587,456.014	~0
Model 13	−743,951,293.5	353,336,542.2	5,214,852.061	~0
Model 14	−743,705,455.6	357,823,698.1	125,487.098	~0
Model 15	−741,996,174.1	353,358,963.1	1,253,698.017	~0
Model 16	−740,495,214.930	356,854,712.2	2,548,756.369	~0
Model 17	−745,012,396.560	359,586,325.2	2,548,741.258	~0
Model 18	−739,818,631.185	356,854,120.5	369,852.369	~0

a Based on the best likelihood among the 50 independent runs for each model.

b The calculation of AIC_i_, Δ_i_ and w_i_ are according to the methods shown in Excoffier et al. (2013).

**Table 2:** Demographic parameters and confidence interval of the best model.

	Point estimation	95% CI^a^	
		Lower	Upper
Parameters		bound	bound
N_e-ANC_	746,525	721,632	756,985
N_e-_*_P.tremula_*	905,400	891,235	912,578
N_e-_*_P.davidiana_*	1,893,583	1,883,565	1,902,325
m*_P.tremula_*_->_ *_P.davidiana_*	4.43 × 10^−8^	4.02 × 10^−8^	4.68 × 10^−8^
m*_P.davidiana_*_->_ *_P.tremula_*	2.52 × 10^−7^	2.35 × 10^−7^	2.69 × 10^−7^
TDIV	3,600,865	3,586,125	3,652,369

**Figure 5:**
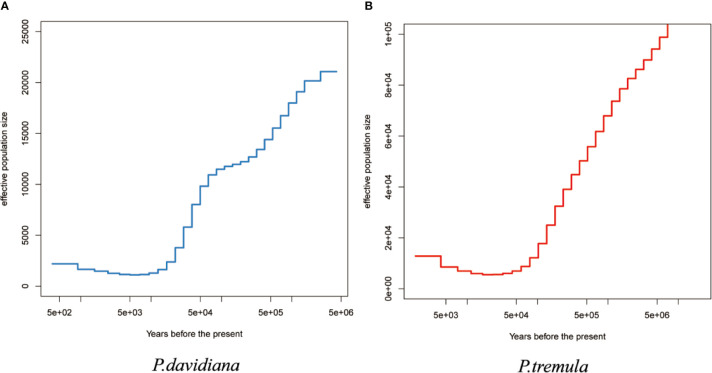
The effective population size of **(A)** *P. davidiana* and **(B)** *P. tremula* (Ne) over historical time implementing by MSMC.

Parameters are defined in **Figure 5**. N_e-ANC_, N_e-N_, N_e-P.tremula_ and N_e-P.davidiana_ indicate the effective population sizes of ancestral, P. tremula and P. davidiana population respectively, m_P.tremula__->__P.davidiana_ indicates the per generation migration rate from P. tremula to P. davidiana, m_P.davidiana__->__P.tremula_ indicates the per generation migration rate from P. davidiana to P. tremula, TDIV indicates the estimated divergence time between P. tremula and P. davidiana populations.

a Parametric bootstrap estimates obtained by parameter estimation from 100 data sets simulated according to the overall maximum composite likelihood estimates shown in point estimation columns. Estimations were obtained from 100,000 simulations per likelihood.

The effective population size (*Ne*) over historical time was also evaluated in the *P. davidiana* and *P. tremula* populations. Higher resolution of recent population size changes is expected when more haplotypes are used (Schiffels and Durbin, 2014). Four individuals and eight haplotypes were used to infer changes in *N*_e_ for each population. Additional numbers were not used so as to limit computing cost. The two species experienced considerably long periods of bottleneck following divergence. Population expansion in *P. tremula* occurred around 10,000–20,000 years ago and continued up to the present (**Figure 5B**), whereas *P. davidiana* experienced a population expansion following a substantially longer periods of bottleneck (**Figure 5A**).

### Genome Differentiation and Identification of Outlier Regions

*F_ST_* was calculated between *P. davidiana* and *P. tremula* using 10,000 bp windows to investigate the genetic differentiation patterns across the genome. The fixation index *F_ST_* is a standard genetic differentiation parameter and therefore sensitive to any process that alters interspecific variation. In the present study, the genetic differentiation coefficient *F_ST_* was calculated for the two species. We found that genetic differentiation was evident between the two populations, The *F_ST_* values between *P. davidiana* and *P. tremula* was 0.3625 (**Table 4**). We also calculated dxy, total sequence differentiation between the populations, an absolute criterion for evaluation of interspecific differentiation. Sequence differentiation was also evident among the two populations, with dxy values between *P. davidiana* and *P. tremula* found to be 0.2511 (**Table 4**).

**Figure 6:**
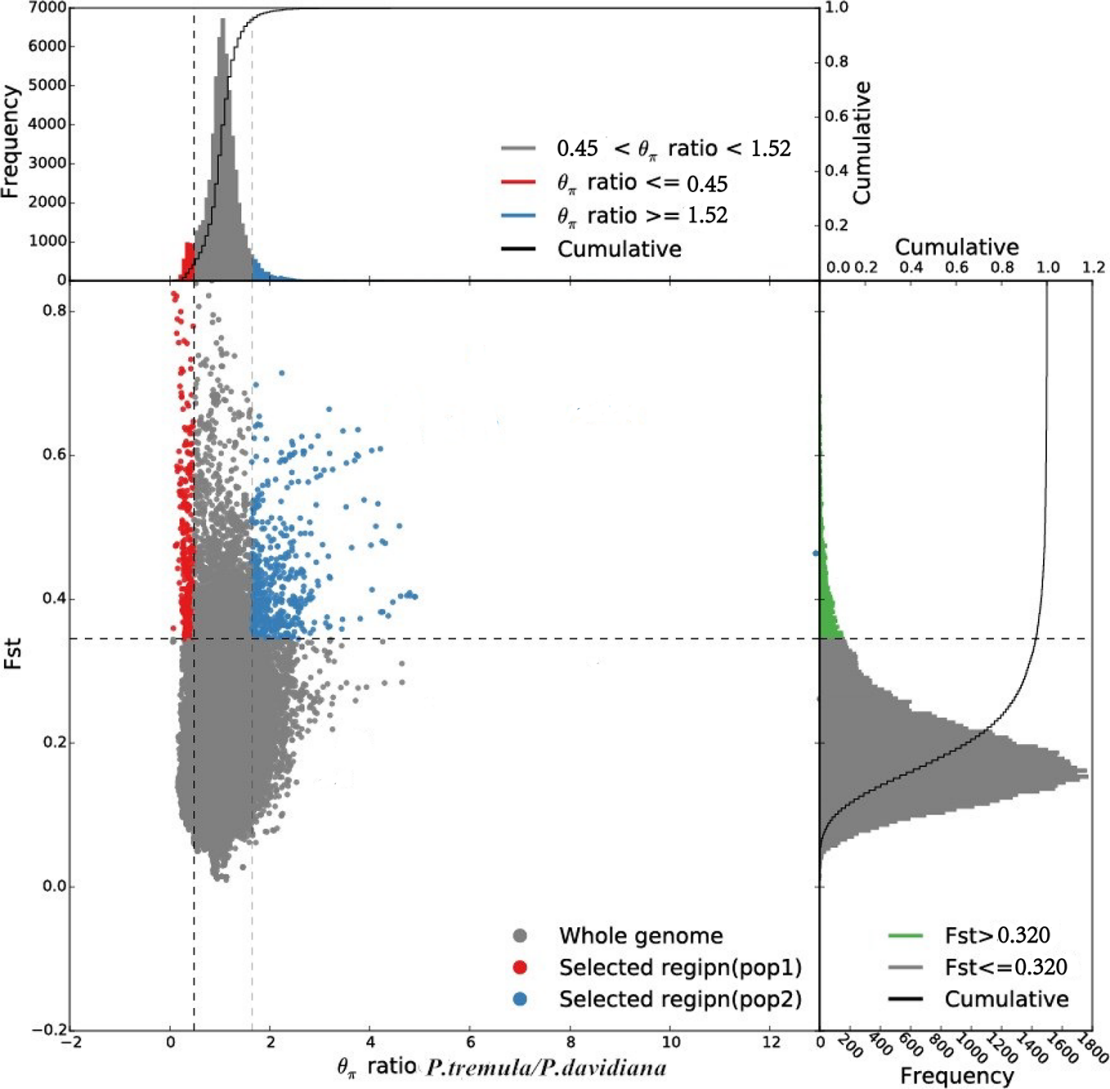
Distribution of θπ ratios (*P. tremula*/*P. davidiana*) and *F_ST_* values, which are calculated in 100 kb windows sliding in 10 kb steps. Data points located to the left and right of the left and right vertical dashed lines, respectively (corresponding to the 5% left and right tails of the empirical θπ ratio distribution), and above the horizontal dashed line (the 5% right tail of the empirical *F_ST_* distribution) were identified as selected regions for the *P. tremula* (red points) and *P. davidiana* (blue points) populations.

Comparing the empirical distribution of inter-specific *F*ST with that obtained from simulations based on the best-fitting demographic model, we found that the empirical distribution was flatter and contained greater proportions of regions falling in the extremes of distribution (**Figure 6**). We also identified the top 1% of *F_ST_* values and the negative end of Tajima’s D values were selected as highly differentiated regions with a selective sweep (Chen et al., 2018) and detected a poorly differentiated region with an *F_ST_* value of less than 0.15. We identified 310 highly differentiated regions and 680 that were poorly differentiated (False Discovery Rate <0.01), randomly distributed throughout the genome, these outlier windows possibly affected by natural selection.

### Population Genetic Analysis

A large number of inspectable neutral loci and evolutionary information were contained in the genome. This was also valuable as an important reference to ascertain whether the *P. davidiana* population was the center of adaptability and diversification. Throughout the genome, we observed that the genetic diversity parameters π, θ_W_, and H_E_ of the *P. davidiana* population were highest, and the *P. tremula* population had the lowest genetic diversity (**Table 4**). Tajima’s D parameters of the *P. davidiana* population was >0, and that of the *P. tremula* population <0. The recombination rate ρ of the *P. davidiana* population was much higher than that of *P. tremula* population (**Table 4**).

Genome-wide linkage disequilibrium (LD) also varied markedly; specifically, the average distance over which LD decayed to half of its maximum value. The *P. davidiana* and *P. tremula* populations exhibited different LD decay curves (**Figure S4**), suggesting that the demographic histories of the two species was diverse. The LD pattern of the genome may be altered by population reduction or genetic differentiation. The *P. davidiana* population possessed the smallest LD value and fastest decay rate, while the *P. tremula* population had the largest LD value and slowest decay rate (**Figure S4**).

### Signatures of Selection in Outlier Regions and Effect of Recombination Rate

**Figure 7:**
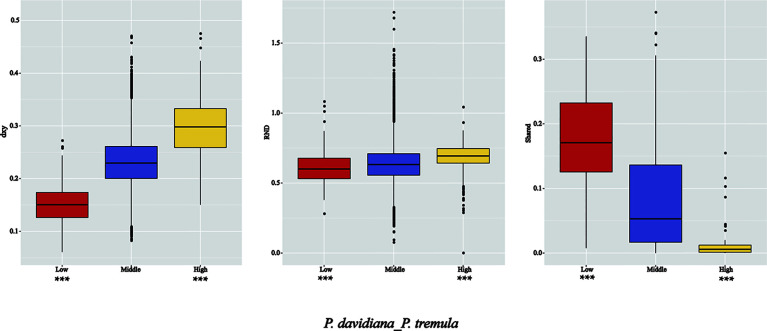
Comparisons of dxy, RND and shared among regions displaying significantly high (yellow boxes) and low (red boxes) differentiation versus the genomic background (blue boxes) between *P. davidiana* and *P. tremula*.

**Figure 8:**
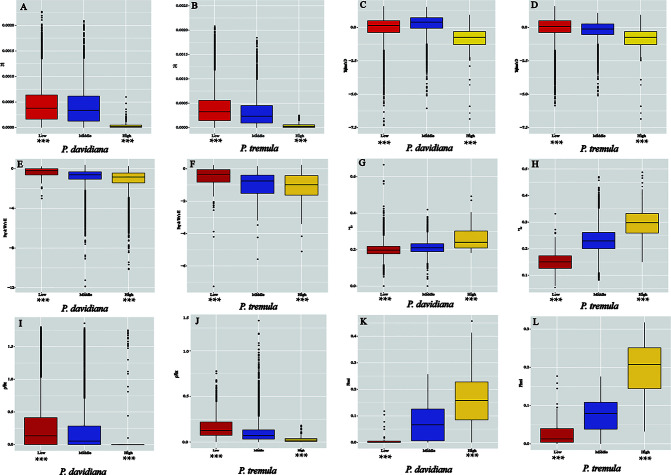
The outlier regions that have been tested to be significantly influenced by natural selection. **(A, B)** Comparisons of nucleotide diversity π among regions displaying significantly high (yellow boxes) and low (red boxes) differentiation versus the genomic background (blue boxes) between *P. davidiana* and *P. tremula*.; **(C, D)** Comparisons of Tajima’s D in *P. davidiana* and *P. tremula*; **(E, F)** Comparisons of Fay & Wu’s H in *P. davidiana* and *P. tremula*; **(G, H)** Comparisons of r^2^ in *P. davidiana* and *P. tremula*; **(I, J)** Comparisons of recombination rate (ρ/θ_π_) in *P. davidiana* and *P. tremula*; **(K, L)** Comparisons of the proportion of fixed differences caused by derived alleles fixed in *P. davidiana* and *P. tremula*. Asterisks designate significant differences between outlier windows and the rest of genomic regions by Mann–Whitney U test (*P-value <0.05; **P-value <1e−4; ***P-value <2.2e−16).

As *F_ST_* is a relative measure of differentiation and is thus sensitive to any processes that alter intra-species genetic variation, we quantified and compared inter-specific genetic differentiation between two unions of outlier windows and the rest of the genome using several additional approaches (**Table 3**). The RND and dxy values of the highly differentiated regions between the two populations showed significantly greater differentiation compared with regions of low differentiation and, in accordance with these patterns, the proportion of inter-specific shared polymorphisms was significantly lower in these regions (**Figure 7**). We also found that highly differentiated regions of the two populations are characterized by multiple signatures of positive selection (Nielsen, 2005). For example, the level of polymorphism (π) of both *P. davidiana* and *P. tremula* populations were extremely low (**Figures 8A, B**). The more negative Tajima’s D values revealed rare alleles that appeared frequently (**Figures 8C, D**), whereas the more negative Fay & Wu’s H demonstrated derived alleles that appeared frequently (**Figures 8E, F**). A more apparent feature was the highly differentiated regions with stronger signals of linkage disequilibrium (LD) (**Figures 8G, H**) (P <0.001, Mann–Whitney U test). We also compared alleles fixed in the *P. davidiana* and *P. tremula* populations and inter-specific shared polymorphisms between the two populations. The results indicated that the proportion of inter-specific shared polymorphisms in the highly differentiated regions was extremely low and the proportion of fixed differences significantly high in both the *P. davidiana* and *P. tremula* populations (**Figures 8K, L**).

**Table 3:** Summary statistics comparing regions displaying extreme genetic differentiation with the rest of the genomic regions in both *P. tremula* and *P. davidiana* (the mean ± standard deviation values are shown).

Parameters	Species	Regions diplaying high differentiation	Regions diplaying low differentiation	Background
θ_π_	*P. tremula*	0.0075 (± 0.0047)^***^	0.0355 (± 0.0165)^***^	0.0146 (± 0.0078)
	*P. davidiana*	0.0093 (± 0.0055)^***^	0.0357 (± 0.0162)^***^	0.0162 (± 0.0078)
Tajima’s D	*P. tremula*	−0.9286 (± 0.5202)^***^	−0.0661 (± 0.5328)^***^	−0.2995 (± 0.5083)
	*P. davidiana*	−1.6588 (± 0.4178)^***^	−0.6058 (± 0.5533)^***^	−1.1554 (± 0.4561)
Fay & Wu’s H	*P. tremula*	−0.6800 (± 0.3885)^***^	−0.1172 (± 0.2596)^***^	−0.3985 (± 0.2795)
	*P. davidiana*	−0.5153 (± 0.3024)^***^	−0.1145 (± 0.2452)^***^	−0.3223 (± 0.2195)
r^2^	*P. tremula*	0.2885 (± 0.1405)^***^	0.1986 (± 0.0965)	0.2115 (± 0.1205)
	*P. davidiana*	0.2482 (± 0.1372)^***^	0.1663 (± 0.0958)^*^	0.1578 (± 0.1110)
ρ/θ_π_	*P. tremula*	0.1627 (± 0.2634)^***^	0.1140 (± 0.1292)^**^	0.2396 (± 0.3533)
	*P. davidiana*	0.2596 (± 0.3536)^***^	0.2088 (± 0.2389)^**^	0.5385 (± 0.5223)
Fixed (%)	*P. tremula*	0.0569 (± 0.0326)^***^	~0 (± 0.0000)^***^	0.0056 (± 0.0098)
	*P. davidiana*	0.0432 (± 0.0292)^***^	~0 (± 0.0000)^***^	0.0036 (± 0.0075)
Shared (%)		0.0789 (± 0.0339)^***^	0.3465 (± 0.0937)^***^	0.1679 (± 0.0628)
*F_ST_*		0.7301 (± 0.0439)^***^	0.1123 (± 0.0182)^***^	0.3805 (± 0.1215)
d_xy_		0.3023 (± 0.0047)^***^	0.0402 (± 0.0181)^***^	0.0250 (± 0.0112)
RND		0.7312 (± 0.2015)^***^	0.6885 (± 0.1972)^***^	0.5517 (± 0.1432)

Asterisks designate significant differences between the regions displaying exceptionally genetic differentiation and the rest of genomic regions by Mann–Whitney U test (*P-value <0.05; **P-value <1e−4; ***P-value <2.2e−16).

In contrast to patterns found in regions of high differentiation, regions of low differentiation had long-term balancing selection characteristics (Charlesworth et al., 1995). For example, the RND and dxy values of the regions that were poorly differentiated between the two populations exhibited less differentiation compared with regions exhibiting high differentiation (**Figures 7**), regions of low differentiation showed significantly higher levels of polymorphism (π) in both *P. davidiana* and *P. tremula* populations (**Figures 8A, B**). The higher Tajima’s D and Fay & Wu’s H parameters revealed intermediate-frequency alleles that appeared frequently (**Figures 8C–F**). Consistent with this prediction, we found slightly lower or comparable levels of LD in these regions (**Figures 8G, H**), possibly influenced by recombination (Lee et al., 2011). The proportion of inter-specific shared polymorphisms in the poorly differentiated regions was higher (**Figure 7**) and the proportion of fixed differences negligible in both the *P. davidiana* and *P. tremula* populations (**Figures 8K, L**).

Because ρ = 4*N_e_*c, where c is the per-generation recombination rate and *N_e_* is the effective population size, a reduction of *N_e_* in regions linked to selection will lower local estimates of ρ even if local c is identical to other regions in the genome. Therefore, recombination rate is also an important factor affecting genome differentiation. To eliminate this effect, we evaluated the effect of recombination rate on genomic differentiation by calculating ρ/θ_π_ with extreme genetic differentiation and the remainder of the genome. In particular, we found a significant negative correlation between *F_ST_* and the rate of recombination. The rate of recombination of the highly differentiated regions was extremely low, with a poorly differentiated region with a higher recombination rate (**Figures 8I, J**).

## Discussion

Understanding how and why genomes diverge during speciation is fundamental to an understanding of how species evolve. With the advance of high-throughput sequencing technologies, considerable progress has been made in documenting the genomic landscape of divergence between recently evolved species. We use a population genomic approach to resolve the evolutionary histories of *P. davidiana* and *P. tremula* and to highlight how genome-wide patterns of differentiation have been influenced by a variety of evolutionary processes. We found that the two aspen were roughly divided into two groups according to their ecological characteristics and geographical distribution: *P. davidiana* and *P. tremula*. We calculated *F_ST_* and dxy values across the genome and found that there was clear genetic differentiation among the two populations.

### Demographic History of the Two Aspen Species

Our analyses indicated that the ancestors of the two aspen diverged into *P. davidiana* and *P. tremula* populations approximately 3.60 million years ago (Mya) (**Figure 5**). The divergence time of *P. davidiana* and *P. tremula* was dated to have occurred during the late Miocene to early Pliocene (c. 4.18 Ma, 95% HPD 0.33–8.35 Ma). The divergence time frame of *P. tremula* and *P. davidiana* was also in accordance with the rapid uplift of Qinghai–Tibet Plateau (QTP) around the Miocene/Pliocene boundary (Long et al., 2013). Historical geology and climatic oscillations especially can shape the geographical distributions patterns of a lot of plant species and triggered population differentiation and even speciation (Hewitt, 2004; Qiu et al., 2011). During the Quaternary and Pliocene periods, dramatic climatic oscillations and historical geology events have caused the uplift of the QTP about 3,000 m (Shi, 2002). During the Quaternary, the global temperatures dropped sharply and the Qinghai–Tibet Plateau (QTP) generally uplifted, so some researchers thought that the most of temperate plants originated mainly from the QTP and its adjacent plateau (Zhang et al., 2004; Kadereit et al., 2008; Wang et al., 2014). Quaternary climate fluctuations and regional uplift easily resulted in geographic isolation among different populations (Han et al., 2017), our research supported the geographic barriers, may have caused the discontinuous distribution pattern of *P. tremula and P. davidiana* where their ancestral population began differentiation due to vicariance. The geographic barriers may have caused the discontinuous distribution pattern of *P. tremula and P. davidiana* where their ancestral population began differentiation due to a vicariance and our research supported the geographic barriers hypothesis. First, dramatic climatic oscillations and historical geology events, such as the Quaternary glaciation and the uplift of the QTP, and the geographic barriers have separated *P. tremula and P. davidiana* into isolated continents that would have impeded the gene flow between *P. tremula* and *P. davidiana* to negligible levels. For example, we found that genetic differentiation was evident between the two populations, the *F_ST_* values between *P. davidiana* and *P. tremula* was 0.3625 (**Table 4**) and the gene flow between *P. davidiana* and *P. tremula* was considerably low (4.43 × 10^−8^ and 2.52 × 10^−7^) (**Figure 5**), which further proved that gene flow between *P. tremula* and *P. davidiana* was impeded to insignificant levels because of the existence of geographic barriers. Moreover, the uplift of QTP and climate oscillations (such as the existence of glacial refugia) could have fragmented the distribution of the ancestral population of *P. davidiana* and *P. tremula* and driven the speciation divergence. Geographical isolation had impeded gene flow between the populations (Hancock and Bergelson, 2011). An universal interpret about biogeographic pattern indicates that the majority of these genera originated mainly from the QTP and its adjacent plateau, and due to historical tectonism and climate oscillations migrated to other regions where they triggered divergence and speciation (Zhang et al., 2007; Zhang et al., 2010). At the same time, different selection pressures on different populations caused the isolated population to gradually accumulate genetic variation, resulting in differentiation between *P. tremula* and *P. davidiana*. Given the modern-day geographic isolation, disjunct distribution and extremely low rates of gene flow, our results support an allopatric model of speciation for these two aspen species (Morjan and Rieseberg, 2004).

**Table 4:** Mean (± standard deviation) values of population genomic statistics (θ_π_, θ_w,_ H_E,_ Tajima’s D, ρ, *F*_ST_ and dxy) comparisons between *P. tremula* and *P. davidiana* population.

Species	θ_π_	θ_w_	H_E_	Tajima’s D	ρ	*F_ST_*	d_xy_
*P. tremula*	0.0092	0.0163	0.0285	−0.04	2.85	0.3625	0.2511
*P. davidiana*	0.0085	0.0125	0.0196	0.12	1.20		

The coalescent-based, intra-specific demographic analyses using MSMC demonstrate that the two populations experienced a considerable long-term bottleneck after divergence, with population expansion beginning approximately 20,000 years ago after the end of the last glacial maximum (LGM). This demographic is consistent with many other forest trees in Eurasia (Hewitt, 2000; Hewitt, 2004). Moreover, the current effective population of the *P. tremula* was smallest, and we speculate that the evolutionary force generated by this small population size in the formation of LD is strongest (Strasburg et al., 2012).

### Genomic Differentiation of *P. davidiana* and *P. tremula*

Consistent with the expectation for allopatric speciation, where the absence of gene flow allowed for the accumulation of inter-specific differentiation due to stochastic genetic drift (Chen et al., 2018). We detected a large number of genomic differentiation regions between the populations of the two species. Although the majority of these can be explained by neutral processes (Strasburg et al., 2012), some outlier regions were significantly influenced by natural selection (Nielsen, 2005). Local rates of recombination interact with natural selection and are known to have a profound effect on patterns of genomic diversity (Cutter and Payseur, 2013). The *F_ST_* value would be expected to be high in those regions with a low recombination rate if natural selection was the principal evolutionary factor for genetic differentiation of the populations of the two species (Noor and Bennett, 2009), because natural selection, such as selective sweeps and background selection remove neutral variation, especially in areas with very low recombination rates (Begun et al., 2007). Accordingly, relative measures of divergence (*F_ST_*) and absolute divergence (dxy) will be higher, depending on intraspecific genetic diversity in areas with lower rates of recombination (Noor and Bennett, 2009; Nachman and Payseur, 2012). Consistent with the observations above, we found a significant negative correlation between *F_ST_* and recombination rate (ρ) in both *P. davidiana* and *P. tremula* populations. As a consequence, our results highlight that linked selection and ρ were important factors of genomic differentiation between *P. davidiana* and *P. tremula* populations (Cruickshank and Hahn, 2014). Our findings thus highlight significant effects of linked selection and recombination in generating the heterogeneous differentiation landscape we observe between the two *Populus* species (Turner et al., 2005; Burri et al., 2015). The long-term action of linked selection in ancestral as opposed to extant lineages can also affect the amount and distribution of ancestral polymorphisms (Ma et al., 2018), which can further result in heterogeneous patterns of genealogical relationships among closely related species (Mailund et al., 2014).

The highly differentiated regions in the present study did not cluster into large regions of the genome (Cruickshank and Hahn, 2014), but into narrow differentiation islands throughout the genome. The majority of the islands were located in regions with limited recombination. Linked selection included positive selection (advantageous mutations) and purifying selection (deleterious mutations), which are also referred to as genetic hitchhiking and background selection (Turner et al., 2005; Noor and Bennett, 2009; Cruickshank and Hahn, 2014). Therefore, we evaluated numerous population genetic parameters to understand how genomic variation occurred during population differentiation and how diverse evolutionary forces drove the differentiation of the entire genome in *P. davidiana* and *P. tremula* populations. We found significant characteristics of positive selection in the populations of the two species (Nielsen, 2005).

For example, the level of polymorphism (π) in both *P. davidiana* and *P. tremula* populations was extremely low. Particularly in the absence of gene flow, selection due to, for instance, local ecological adaptation can result in reduced within-population diversity and indirectly inflate *F_ST_* (Cruickshank and Hahn, 2014). The more negative Tajima’s D revealed that rare alleles appeared frequently, whereas the more negative Fay & Wu’s H indicated that derived alleles appeared frequently. Under a selective sweep model, genetic variants linked to beneficial mutations acted upon by positive selection hitchhike along and reach high frequency (Kaplan et al., 1989). A more apparent feature was that the highly differentiated regionaspens and poplars (Lin et al., 2018). Therefore, although the role of background selection cannot be completely ignored, it is clear that positive selection was the principal evolutionary force driving the differentiation of *P. davidiana* and *P. tremula* genomes. Under the process of positive selection, although genetic diversity was reduced, interspecific differentiation increased. Earlier speciation of *Populus* studies have revealed that apart from background selection, recent positive selection and long-term balancing selection have also been crucial components in shaping patterns of genome-wide variation during the *Populus* speciation process (Wang et al., 2020). However, since it is difficult to accurately estimate the variation in these highly differentiated regions exhibiting low genetic diversity, more caution is required in interpreting the functional characteristics of the overrepresented genes identified here. Therefore, more in-depth research is required on these functional genes in order to clarify how widespread forest tree species respond to climate change during adaptive evolution.

In addition to the characteristics of positive selection, being found in the highly differentiated regions, long-term balancing selection was also identified in the poorly differentiated regions in both species populations (Charlesworth et al., 1995). For example, absolute interspecific divergence (dxy and RND values) was lower than in the highly differentiated regions. The genetic diversity (π) of both *P. davidiana* and *P. tremula* populations was significantly high. In comparison to purifying and positive selection, long-term balancing selection favors the maintenance of advantageous polymorphisms for many generations, which instead result in genomic regions with elevated genetic diversity and reduced *F_ST_* (Hittinger et al., 2010). Higher Tajima’s D and Fay & Wu’s H values revealed that intermediate-frequency alleles appeared frequently, with levels of LD lower than the highly differentiated regions, which may have been influenced by recombination (Lee et al., 2011). The proportion of inter-specific shared polymorphisms in the poorly differentiated regions was higher and the proportion of fixed differences negligible in both *P. davidiana* and *P. tremula* populations. Nevertheless, some caution should still be applied when interpreting these results, because our analyses using a single *P. trichocarpa* reference genome for multiple-species comparisons inevitably suffers from over-representations of conserved genic regions and under-representations of repeat-rich regions as well as other intergenic regions. Future studies therefore need to explore whether the same pattern can be found in complex, repetitive genomic regions in *Populus* and other species.

## Conclusions

Here we provide insights into the speciation and recent evolutionary histories of two closely related forest tree species, *P. tremula* and *P. davidiana*. Our study supports an allopatric model of speciation for the two species. The study indicated an evident genetic differentiation between the two species, if fact, the *F_ST_* values between *P. davidiana* and *P. tremula* was 0.3625. The ancestors of the two aspen diverged into *P. davidiana* and *P. tremula* species approximately 3.60 million years ago (Mya), which was in accordance with the rapid uplift of Qinghai–Tibet Plateau (QTP) around the Miocene/Pliocene boundary. The two species experienced a considerable long-term bottleneck after divergence, with population expansion beginning approximately 20,000 years ago after the end of the last glacial maximum. Coalescent simulations suggest that stochastic genetic drift and historical demographic processes can largely explain the genome-wide patterns of differentiation between species. However, there is an excess of regions displaying extreme inter-specific genetic differentiation in the observed data compared with demographic simulations. We infer that heterogeneous genomic divergence is strongly driven by linked selection and variation in recombination rate in the two species. Instead of being clustered into a few large genomic “islands” as is expected under a model of speciation with gene flow, regions of pronounced differentiation are characterized by multiple signatures of positive selection in both species, and are distributed throughout the genome at many small, independent locations. Regions displaying exceptionally low differentiation are likely candidate targets of long-term balancing selection. Our research highlights that more information need to be integrated into future work when interpreting genomic variation during speciation. These include strict neutral theory, demographic fluctuations, genetic drift, geographical isolation, gene flow, sources of adaptation, positive selection (advantageous mutations) and purifying selection (deleterious mutations).

## Data Availability Statement

The datasets presented in this study can be found in online repositories. The names of the repository/repositories and accession number(s) can be found below: http://bigd.big.ac.cn/gsa, CRA001674 and CRA001683.

## Author Contributions

ZH performed the experiments and wrote the study. AL designed the research.

## Funding

Financial support for this research was provided by the Fundamental Research Funds of China West Normal University (Grant No. 19E044) and the Open Fund of State Key Laboratory of Tree Genetics and Breeding (Chinese Academy of Forestry) (Grant No. TGB2019002).

## Conflict of Interest

The authors declare that the research was conducted in the absence of any commercial or financial relationships that could be construed as a potential conflict of interest.
