# Supplementary figures and images for: Population Genomics Reveals Demographic History and Genomic Differentiation of Populus davidiana and Populus tremula
Source: Front Plant Sci. 2020 Jul 22;11:1103. doi: 10.3389/fpls.2020.01103 (PMC7396531; doi:10.3389/fpls.2020.01103)

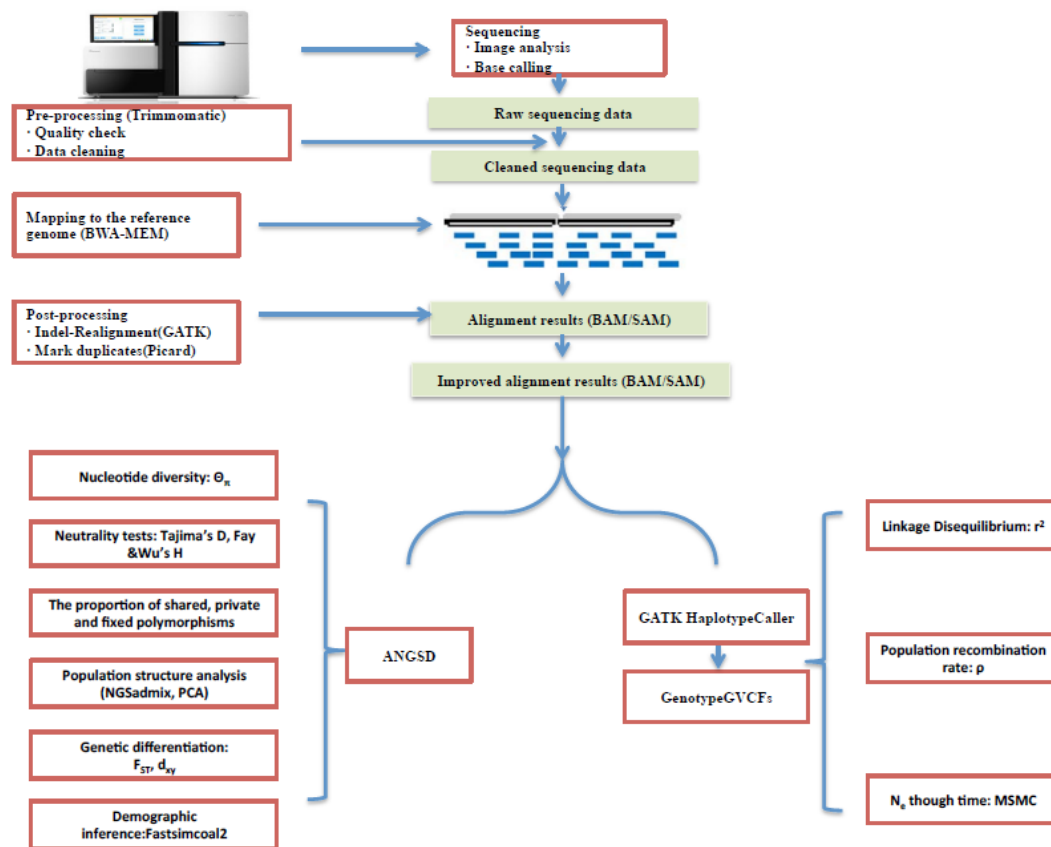

**Figure S1.** Analysis workflow in this study  
(J. Wang, Street, Scofield, & Ingvarsson, 2016)

Supplement: Supplementary file 1 [file DataSheet_1.pdf]
