# Supplementary material for: Population Genomics Reveals Demographic History and Genomic Differentiation of Populus davidiana and Populus tremula
Source: Front Plant Sci. 2020 Jul 22;11:1103. doi: 10.3389/fpls.2020.01103 (PMC7396531; doi:10.3389/fpls.2020.01103)
Supplement: Supplementary file 2 [file DataSheet_2.pdf]

Model 1

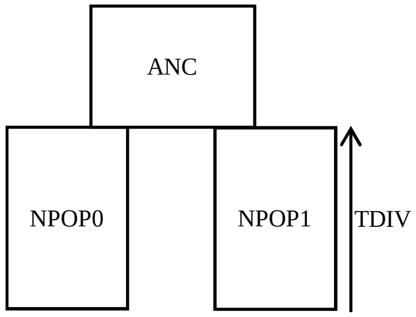

Model 2

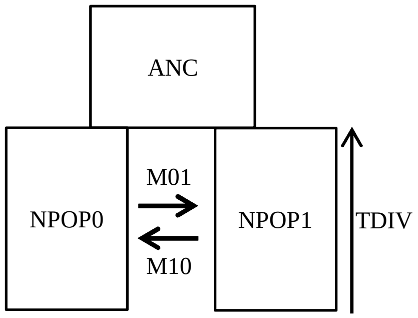

Model 3

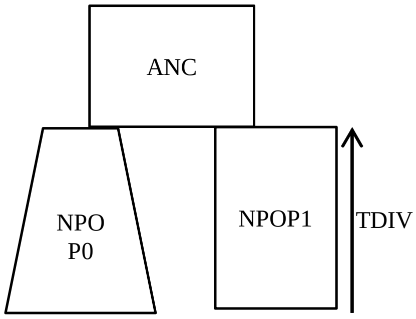

Model 4

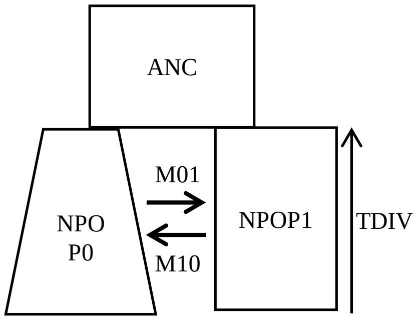

Model 5

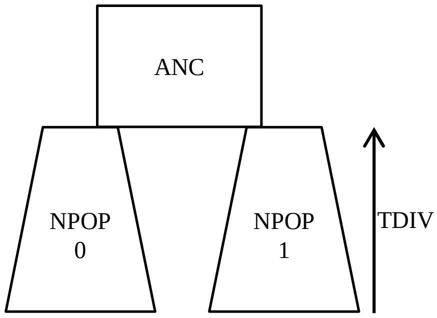

Model 6

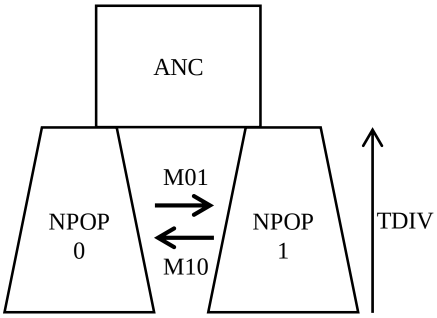

Model 7

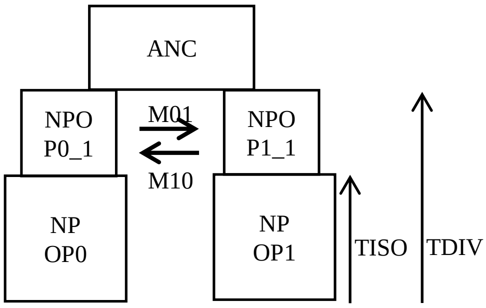

Model 8

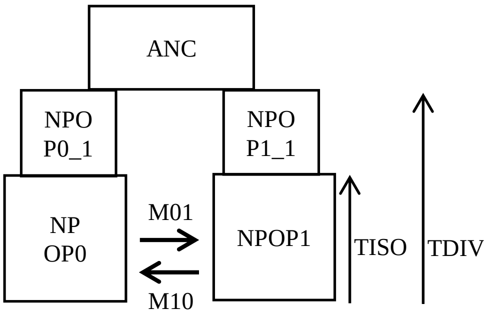

Model 9

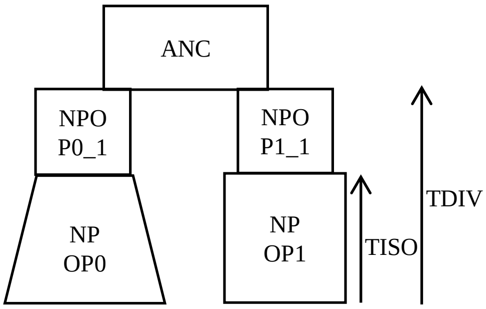

Model 10

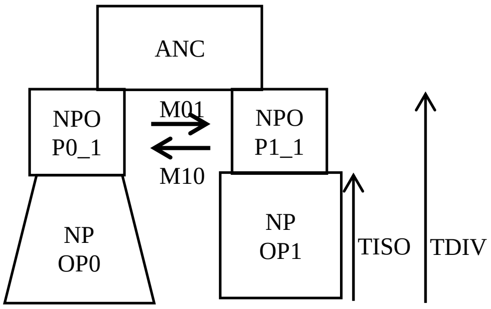

Model 11

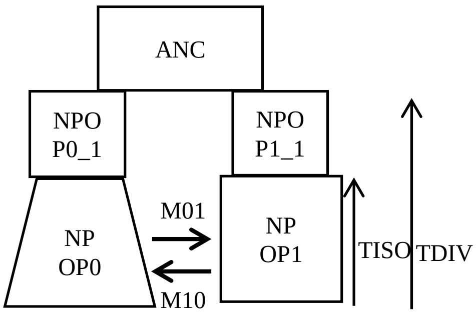

Model 12

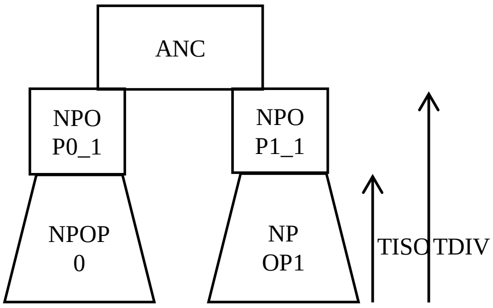

Model 13

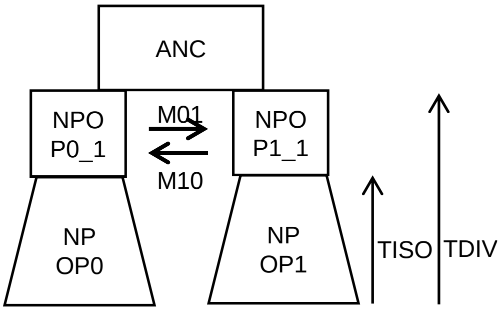

Model 14

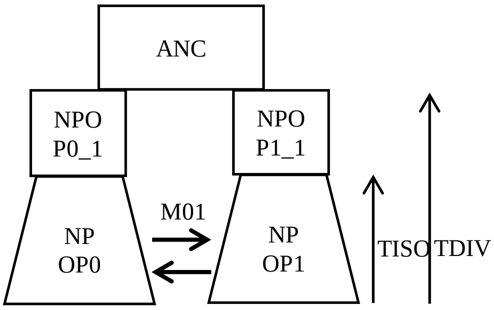

Model 15

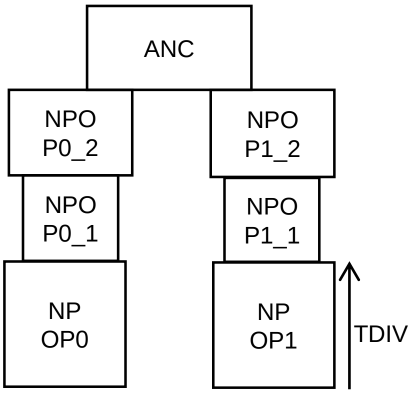

Model 16

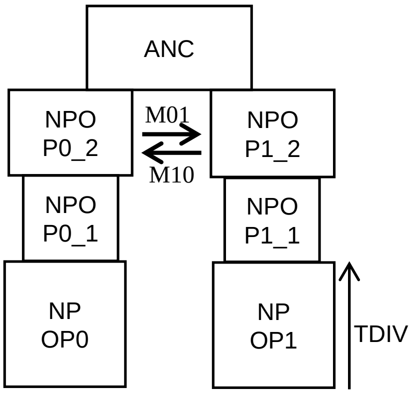

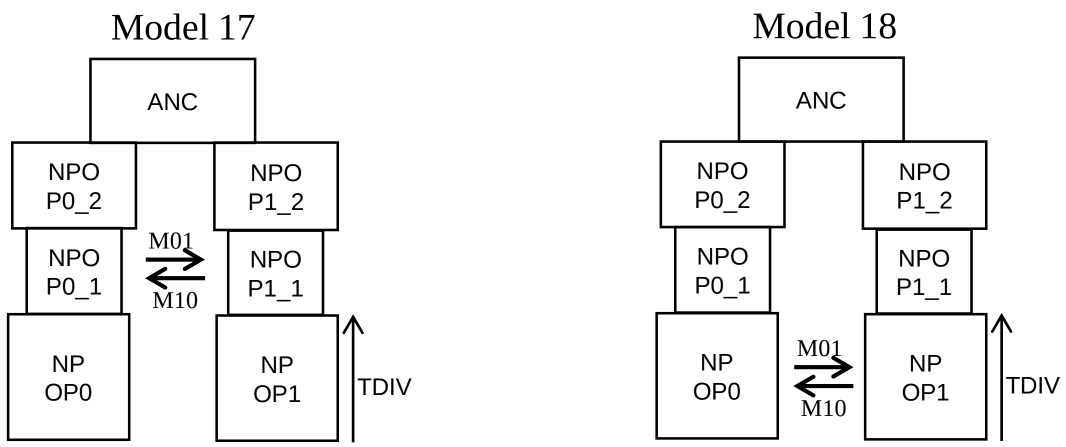

Figure S2. Tested demographic models. Model1, isolation of two species without gene flow; model2, isolation of two species with asymmetric gene flow; model3, isolation of two species with exponential population size change in *P. tremula* and stepwise population size change in *P. davidiana*, no gene flow; model4, isolation of two species with exponential population size change in *P. tremula* and stepwise population size change in *P. davidiana*, with asymmetric gene flow; model5, isolation of two species with exponential population size changes in both species, no gene flow; model6, isolation of two species with exponential population size changes in both species, with asymmetric gene flow; model7, isolation of two species with stepwise population size changes in both species, asymmetric gene flow in the early stage of species divergence until the time of TISO, no gene flow afterwards; model8, isolation of two species with stepwise population size changes in both species, no gene flow in the early stage of species divergence until the time of TISO, asymmetric gene flow afterwards; model9-model11, isolation of two species with two steps of population size changes in both species, both species experienced stepwise population size changes until the time of TISO, afterwards, *P. tremula* experienced exponential population size change, and *P. davidiana* experienced another stepwise change, the difference between models is the occurrence and the time of gene flow between species; model12-model14, isolation of two species with two steps of population size changes in both species, both species experienced stepwise population size changes until the time of TISO, afterwards, both species experienced exponential population size changes, the difference between models is the occurrence and the time of gene flow between species ; model 15-model18, isolation of two species with three steps of stepwise population size changes in both species, the difference between models is the occurrence and the time of gene flow between species.
