# Supplementary material for: Population Genomics Reveals Demographic History and Genomic Differentiation of Populus davidiana and Populus tremula
Source: Front Plant Sci. 2020 Jul 22;11:1103. doi: 10.3389/fpls.2020.01103 (PMC7396531; doi:10.3389/fpls.2020.01103)
Supplement: Supplementary file 3 [file Table_1.docx]

**Table S1.** Summary statistics of Illumina re-sequencing data per sample

| **SampleID** | **Latitude** | **Longitude** | **Mapping rate (%)** | **Mean Coverage** |
| --- | --- | --- | --- | --- |
| ***P. tremula*** |  |  |  |  |
| *P. tremula*1 | E 56°69′ | N 13°21′ | 91.34% | 20.50 |
| *P. tremula*2 | E 56°73′ | N 13°25′ | 90.52% | 23.70 |
| *P. tremula*3 | E 56°34′ | N 15°02′ | 90.06% | 48.58 |
| *P. tremula*4 | E 56°30′ | N 15°12′ | 88.78% | 24.53 |
| *P. tremula*5 | E 57°99′ | N 12°91′ | 89.54% | 18.50 |
| *P. tremula*6 | E 57°98′ | N 12°93′ | 88.62% | 17.25 |
| *P. tremula*7 | E 57°84′ | N 15°32′ | 88.56% | 20.32 |
| *P. tremula*8 | E 57°82′ | N 15°31′ | 90.27% | 20.11 |
| *P. tremula*9 | E 59°64′ | N 12°94′ | 88.90% | 20.25 |
| *P. tremula*10 | E 59°63′ | N 12°96′ | 89.72% | 19.46 |
| *P. tremula*11 | E 59°81′ | N 17°98′ | 88.66% | 22.25 |
| *P. tremula*12 | E 59°77′ | N 17°98′ | 88.43% | 23.88 |
| *P. tremula*13 | E 61°19′ | N 13°80′ | 88.56% | 22.25 |
| *P. tremula*14 | E 61°30′ | N 13°72′ | 88.78% | 22.27 |
| *P. tremula*15 | E 61°71′ | N 16°73′ | 90.34% | 36.74 |
| *P. tremula*16 | E 61°69′ | N 16°67′ | 88.88% | 21.01 |
| *P. tremula*17 | E 64°34′ | N 16°39′ | 90.00% | 24.44 |
| *P. tremula*18 | E 64°33′ | N 16°37′ | 89.94% | 23.22 |
| *P. tremula*19 | E 63°97′ | N 20°70′ | 90.48% | 23.43 |
| *P. tremula20* | E 63°86′ | N 20°49′ | 90.27% | 24.60 |
| *P. tremula21* | E 66°02′ | N 18°57′ | 91.27% | 22.23 |
| ***P. davidiana*** |  |  |  |  |
| P. davidiana1 | E 131°15′ | N 46°64′ | 92.36% | 30.50 |
| P. davidiana2 | E 131°15′ | N 46°64′ | 90.25% | 33.70 |
| P. davidiana3 | E 130°55′ | N 46°23′ | 90.08% | 40.58 |
| P. davidiana4 | E 130°55′ | N 46°23′ | 90.78% | 32.25 |
| P. davidiana5 | E 127°52′ | N 50°24′ | 91.25% | 34.23 |
| P. davidiana6 | E 127°52′ | N 50°24′ | 92.36% | 30.08 |
| P. davidiana7 | E 127°52′ | N 50°24′ | 88.08% | 31.25 |
| P. davidiana8 | E 127°52′ | N 50°24′ | 89.98% | 30.25 |
| P. davidiana9 | E 127°52′ | N 50°24′ | 90.05% | 35.62 |
| P. davidiana10 | E 124°49′ | N 44°47′ | 93.25% | 36.28 |
| P. davidiana11 | E 124°49′ | N 44°47′ | 89.28% | 33.36 |
| P. davidiana12 | E 124°49′ | N 44°47′ | 88.89% | 32.29 |
| P. davidiana13 | E 124°49′ | N 44°47′ | 90.21% | 36.68 |
| P. davidiana14 | E 123°88′ | N 43°68′ | 93.25% | 30.05 |
| P. davidiana15 | E 123°88′ | N 43°68′ | 88.08% | 32.03 |
| P. davidiana16 | E 123°88′ | N 43°68′ | 90.78% | 31.05 |
| P. davidiana17 | E 123°88′ | N 43°68′ | 92.58% | 30.89 |
| P. davidiana18 | E 126°66′ | N 51°72′ | 90.32% | 35.62 |
| P. davidiana19 | E 126°66′ | N 51°72′ | 91.25% | 32.05 |
| P. davidiana20 | E 126°66′ | N 51°72′ | 93.25% | 30.06 |
